# The Impact of Family Factors on Adolescent Intensive Outpatient Psychotherapy Outcomes for Suicidal Thoughts, Behaviors, and Depression

**DOI:** 10.1111/sltb.70104

**Published:** 2026-05-05

**Authors:** Björn Schlier, Aidan G. C. Wright, Giovanna Porta, Edward Hamilton, Kimberly Poling, Kelsey Bero, David Brent, Giana Teresi, Tina R. Goldstein, Aleksandra Kaurin

**Affiliations:** ^1^ Department of Psychology University of Wuppertal Wuppertal Germany; ^2^ Department of Psychology University of Michigan Ann Arbor Michigan USA; ^3^ Eisenberg Family Depression Center University of Michigan Ann Arbor Michigan USA; ^4^ Western Psychiatric Hospital University of Pittsburgh Medical Center Pittsburgh Pennsylvania USA; ^5^ Department of Psychology University of Pittsburgh Pittsburgh Pennsylvania USA

## Abstract

**Background:**

Although they constitute well‐established risk factors for suicidal thoughts and behaviors (STBs), few studies have investigated how family factors such as family cohesion and family conflict influence the trajectory of treatment response among depressed and suicidal adolescents. In this study, we examined the association between baseline family factors and response to an intensive outpatient program (IOP) for STBs in adolescents.

**Methods:**

Participants (*n* = 637) either reported their satisfaction with family cohesion or family conflict levels at baseline and provided weekly self‐reports of depression and STBs throughout IOP treatment. We calculated multi‐level regression models to test for the interaction effects of treatment‐day × family‐factor‐levels and explored further demographic and clinical moderators.

**Results:**

Higher levels of family cohesion correlated with more reduction of STBs over time (*β* = −0.11). Moreover, the effects of family cohesion and family conflict on treatment were moderated by patient age, ethnic minority status, and symptom severity.

**Discussion:**

STBs and depression improved with IOP, with family cohesion partially moderating treatment response. Differences due to family factors were more prominent in older and ethnically minoritized adolescents, and those with more severe symptoms. Future studies should elucidate how family factors co‐vary with changes in STBs during treatment to better understand these effects.

## 1 Introduction

Various familial risk factors are associated with heightened likelihood of suicidal thoughts and behaviors (STBs) in children and adolescents. In addition to immutable factors, such as a family history of psychopathology, risk factors for youth suicidality encompass a variety of modifiable psychosocial factors. These include family conflict in both young children and adolescents (e.g., DeVille et al. 2020; Miller et al. 2012), low parental monitoring in children (DeVille et al. 2020), and insecure parent–child attachment (Wagner et al. 2003). At the same time, there is also evidence for family resilience factors that correlate with lower STB‐prevalence in youths, such as family cohesion (Miller et al. 2012) and adaptability of the family system (Wagner et al. 2003). Numerous studies offer evidence for the significance of these factors through cross‐sectional and time‐lagged associations between family risk and resilience factors and the onset or severity of suicidality in youths. Moreover, it has been shown that family factors predict the re‐emergence of STBs following initial treatment (King et al. 2024). Empirical evidence syntheses on the subject, however (e.g., Carballo et al. 2020; Wagner et al. 2003), converge on the conclusion that comparatively little is known about the temporal sequence and putative causal relationship by which these factors impact youths' STBs from their emergence to treatment and recovery. Because of the pivotal role of the familial social network in child and adolescent life, it stands to reason that psychosocial family factors may moderate the effectiveness of psychotherapy among suicidal youth.

However, few studies empirically investigate how family factors influence the trajectory of psychological treatment for suicidal youth, that is, to what extent risk factors such as family conflict may impede treatment response, or family resilience factors such as cohesion may facilitate it. The limited existing research on the role of family factors on treatment response in this population tentatively suggests that family factors affect the likelihood of benefitting from treatment. Regarding symptoms of depression in general, family interventions have been shown to be effective in youths (e.g., Miklowitz et al. 2021). For STBs in particular, an early study by King and colleagues (King et al. 1997) showed that family functioning predicts treatment adherence to medical and individual psychological intervention in suicidal adolescents following inpatient treatment, implying that the likelihood for a beneficial treatment outcome increases with higher levels of baseline family functioning and decreases with higher levels of baseline family risk factors.

Further, indirect evidence for the role of family factors could be derived from research on perceived burdensomeness, that is, a belief that other people would be alleviated by one's death. Multiple studies showed perceived burdensomeness to be substantially correlated with low family cohesion and high family conflict (e.g., Hunt et al. 2022). In depth analyses of the situations, in which adolescents experience perceived burdensomeness, found parent‐adolescent relationships to be the most frequent context (Hill et al. 2019). Since a secondary analysis of an efficacy trial for family focused interventions to treat suicidality and depression in adolescents indicated that higher perceived burdensomeness predicted limited treatment benefit (Abbott et al. 2019), the connection between perceived‐burdensomeness and family relations could indicate that lower quality of family relations (i.e., low cohesion/high conflict relationships) shares this predictive quality as the distal causal factor that is mediated by perceived burdensomeness.

By contrast, more recent studies have yielded contradictory results regarding the effect of family factors. For instance, another secondary analysis of an efficacy trial involving attachment‐based and supportive family‐focused interventions (Zisk et al. 2019) yielded reverse moderating effects of baseline family functioning: Patients and their caregivers with more uncooperative communication behavior during a baseline diagnostic conflict discussion showed better treatment outcomes. Conversely, certain malleable family risk/resilience factors for suicidality could act as inverse predictors of intervention success. This underscores the need for further investigation into the complexity of family factors, since a more detailed knowledge about their differential effects on treatment outcomes may help to optimize effective treatment allocation.

To date, research on the role of individual family factors such as family cohesion and family conflicts on treatment trajectories and treatment outcomes remains relatively limited, raising the question of their generalizability to different forms of individual treatment. Furthermore, existing research employs relatively simple assessment designs, such as longitudinal pre–post designs, which constrain the depth of information we can gather concerning trajectories of change and inter‐individual differences within these trajectories.

In this study, we employ intensive longitudinal assessments on a weekly basis to track the IOP treatment process over up to 9 weeks using data from the *Services for Teens At Risk* (STAR) center. Our aim is to understand the trajectories of change in STBs in response to an intensive outpatient treatment program for adolescents experiencing STBs. This treatment integrates cognitive‐behavioral therapy and elements of dialectical behavior therapy through a combination of group therapy, individual therapy, and medication management. Our goal is to assess whether established risk and resilience factors, that is, family cohesion and family conflict at the outset of treatment, are associated with the response to IOP treatment over time. We seek to clarify whether these moderating effects mirror associations found in etiological research, with the underlying assumption that lower levels of family conflict and higher levels of family cohesion predict better treatment response (i.e., less/delayed reduction in STBs as well as depression) due to more readily accessible interpersonal support, e.g., to practice and apply therapy content in everyday life. Or whether family factors demonstrate an inverse association similar to the one found for family interventions, wherein patients with high family risk or low family resilience markers show greater improvement over the treatment period, assuming that treatment gains are accelerated when positive change could lead to improvement throughout a wider range of dire starting conditions. Finally, we aimed to identify potential demographic or clinical moderators that could influence the association between family factors and treatment‐success.

## 2 Methods

### 2.1 Sample Recruitment

All participants were enrolled in a specialized IOP for adolescent depression and suicidality. For the duration of the IOP, participants completed self‐report questionnaires for every week they attended the program (Hamilton et al. 2023; Victor et al. 2023). Teens at the ages of 13–18 who require more intensive care than weekly outpatient treatment were eligible for enrolment into the IOP. Eligibility was determined by clinic staff as part of standard clinical practice. Criteria for need for IOP was determined by one or more of the following criteria:

- Severe depressive symptoms (Children's Depression Rating Scale‐Revised > 55);
- Current suicidal ideation and/or recurrent non‐suicidal self‐injurious behavior;
- Functional impairment (Children's Global Assessment Scale < 55).

Participants were included in this analysis when they had at least received one session of IOP in addition to baseline assessment (resulting in a minimum of 2 assessment points per participant). All study procedures were approved by the University of Pittsburgh Institutional Review Board (Protocol Number: 19030078). Participants and a parent/legal guardian provided written informed assent/consent for their clinical data to be used for research purposes in order to be included in this analysis.

### 2.2 Procedure

#### 2.2.1 Intensive Outpatient Program

Teens in the IOP attend treatment 9 h per week (3 h each/3 days); treatment consists of skills group where they are provided with support and learn Cognitive Behavioral Therapy (CBT; Brent et al. 2011) and Dialectical Behavioral Therapy (DBT) skills (Miller et al. 2007). Additionally, IOP includes weekly individual therapy and medication management. The IOP did not include any mandatory treatment component for parents of youths attending the IOP. The treatment centre offered an optional monthly parent psychoeducation group for interested families that included basic information regarding DBT skills. However, only a small fraction of families visited these groups and attendance was not tracked. A typical treatment according to IOP‐protocol consisted of up to 9 weeks of uninterrupted treatment, with no gaps of more than 3 weeks within this period (Bero et al. 2024). The IOP's intensity and components are comparable to other published and disseminated programs for suicidal adolescents (Kennard et al. 2019; Leffler and D'Angelo 2020; Mochrie et al. 2020). Readiness for discharge from IOP to standard outpatient treatment is indicated by measurement‐based sustained improvements in individualized treatment targets (Victor et al. 2023). Following an IOP treatment epoch, patients may step‐down to outpatient weekly or continuation/maintenance treatment. Finally, due to the naturalistic design of this study, participants could be re‐admitted for another iteration IOP following their initial participation. For our analyses, only the assessments from the first treatment epochs are included.

#### 2.2.2 Assessments

Prior to the start of the treatment, participants were extensively assessed using a comprehensive clinical diagnostic protocol by a trained masters‐level clinician to determine presence of current psychiatric disorders (DSM‐5; APA 2013), which included gathering information from the patient as well as their parent (for more information, see Victor et al. 2023). All diagnoses were reviewed with an attending child psychiatrist to establish a diagnostic consensus.

Additionally, participants' current and most severe levels of suicidal ideation and suicidal behavior at baseline were rated on six‐point scales (ideation: 0 = not suicidal, 5 = active suicidal ideation with plan and intent; behavior: 0 = not suicidal, 5 = multiple attempts) adapted from the KSADS depression rating scale suicidality item (Kaufman et al. 2017).

As part of an electronic baseline questionnaire battery (see Victor et al. 2023), participants completed either a validated assessment of family cohesion (Family Satisfaction Scale—FSS; Olson and Wilson 1982) or of family conflict (Conflict Behavior Questionnaire; CBQ; Robin and Foster 1989). Participants also filled out weekly self‐report questionnaires electronically throughout IOP participation as part of standard measurement‐based clinical care at STAR that included self‐report measures of current suicidality and depression.

### 2.3 Materials

#### 2.3.1 STBs

Current levels of STBs were assessed weekly throughout IOP with the Ask Suicide‐Screening Questions (ASQ; Horowitz et al. 2012) tool. The ASQ consists of four items that ask for different forms of suicidal thoughts (e.g., “have you wished you were dead?”) up to the occurrence of suicidal behavior (e.g., “have you tried to kill yourself?”). Although originally conceptualized as a self‐report measure to be delivered verbally, the ASQ has since been successfully adapted for remote screening in virtual clinic settings (Glasner et al. 2024) and administration in online‐ or paper‐pencil form (Christensen‐LeCloux et al. 2021; Rens et al. 2023), although a direct comparison of different delivery modes is still pending.

In line with the treatment‐aim of reducing suicidal risk, weekly ASQ‐derived assessments of STBs were operationalized as a unidimensional construct mirroring risk for suicidality. This approach was based on existing research supporting a continuum of suicidality including passive ideation, active thoughts, intent and planning, and suicidal behavior (e.g., McKeown et al. 1998) and has been implemented as one‐dimensional indicator across analyses of the trial data (e.g., Bero et al. 2024; Kaurin et al. 2024). For each item, participants answered whether the respective type of suicidality applied to them during the last week (0 = “no”, 1 = “yes”). ASQ‐items were answered in self‐report format by the patients with clinicians present during the IOP‐treatments; clinicians reviewed the answers and positive answers were followed up on, mirroring the outline of the ASQ‐screening protocol. A confirmatory factor analysis with all four dichotomous items loading on one factor and diagonally weighted least squares estimator was performed on the baseline assessments of participants. Results indicated overall good model‐fit (CFI = 0.99, RMSEA = 0.075, SRMR = 0.078), with the first three items that ask about passive to active suicidal thoughts and plans showing very high loadings (0.74–0.99) and the fourth item (“have you tried to kill yourself?”) showing a sufficiently high loading (0.42). As primary outcome we calculated a *STBs sum score* based on the dichotomized starting questions for each item (suicidality sum score range 0–4, internal consistency of day 1 assessments: *α* = 0.59). Additionally, a composite score of the first three items (suicidal thoughts, range 0–3: *α* = 0.64) and a dichotomous outcome suicidal behaviors (item 4) were used for stability analyses.

#### 2.3.2 Depression

Weekly depression levels were assessed with the abbreviated version of the Mood and Feelings Questionnaire (MFQ; Angold et al. 1995). The abbreviated MFQ consists of 13 items (e.g., “I cried a lot”) that do not include any items on STB. Items are answered on a three‐point scale (0 = “not true”, 1 = “sometimes”, 2 = “true”), which are summed up to a depression score (range 0–26) with higher scores (0–26) reflecting higher levels of depression (baseline internal consistency: *α* = 0.83).

#### 2.3.3 Baseline Family Factors

Family cohesion was assessed with the 10‐item Family Satisfaction Scale (FSS; Olson and Wilson 1982). The FSS measures the level of satisfaction a family member has with different aspects of familial functioning pertaining to core facets of family cohesion, such as closeness (e.g., “How satisfied are you with…” “…the degree of closeness between family members?”), support (e.g., “…the family members' concern for each other”), time spent together (e.g., “…the amount of time you spend together as a family”) or familial coping (e.g., “…your family's ability to cope with stress”). Adolescents answered on 5‐point Likert scales, ranging from 1 = “very dissatisfied” to 5 = “very satisfied”. In the current sample, internal consistency was excellent (*α* = 0.95). A mean score was calculated for this study with higher scores indicating more satisfaction with family cohesion.

Family conflict was assessed with the 20‐item adolescent version of the Conflict Behavior Questionnaire (CBQ; Robin and Foster 1989). The CBQ measures family communication‐conflict behavior at home (e.g., “my parents seem to be always complaining about me”). Patients answered for each item whether it applied to them (yes/no). The current sample showed an excellent Cronbach's *α* = 0.91. A sum score was calculated, with higher scores indicating more severe levels of conflict.

### 2.4 Strategy for Data Analysis

Before testing for moderation effects of family factors, we determined the optimal functional form of the rate of improvement during IOP. Based on the result that dose–response‐trajectories in psychotherapy in general follow a log‐linear association with negative acceleration (e.g., Stulz et al. 2013), we assumed the most fitting relationship between treatment course (independent variable: treatment day of the respective assessment) and outcomes (STBs or depression, respectively) to be a log‐linear association in multi‐level model of assessments nested in participants. To verify this assumption, we compared the log‐linear fit with models with a linear, quadratic, and cubic association using the AIC and BIC. Further, we visually compared the predicted log‐linear association with the predicted day‐by‐day outcomes from a parallel multi‐level model, in which the independent variable treatment day is treated as a factor (i.e., multi‐nominal estimation of outcomes without any a priori assumption of the nature of their association). Since treatment length was intended to vary between participants and assessment intervals varied due to the exact assessment‐days varying between weeks, all models used the exact number of the day since treatment started to allow for calculation of treatment trajectories in multilevel‐assessments with differing numbers of assessments and different distributions across the treatment time.

To test whether family factors at the beginning of treatment affected treatment trajectories, we next calculated multi‐level regression models with two independent variables—treatment day and one family factor (i.e., FSS‐score or CBQ‐score)—and one of the outcome variables (ASQ sum score, MFQ depression score) as the dependent variable. Family factor variables were grand‐mean centred and interaction effects of session number × family factor were examined.

For the subsequent analyses of moderation effects, multilevel‐models with a three‐way interaction of treatment day × mean‐centred family factor (FSS or CBQ) × one demographic (e.g., gender, age, ethnicity) or clinical variables indicative of symptom severity (e.g., most severe levels of suicidal thoughts prior to treatment) were tested. Since sufficient power for cross‐level interactions in general require a minimum of 100–200 participants with approximately 10 assessments per participants (Hox 2010), we decided to calculate all models with random intercept and fixed slopes and estimated interaction effects as fixed effects.

All moderation analyses for suicidality were first calculated with the four item ASQ‐sum‐score as the dependent variable. As an exploratory stability analysis, we also re‐calculated the analyses with a three‐item ASQ sum score for suicidal thoughts and within logistic multilevel regression models using the ASQ suicidal behaviors item as the outcome.

## 3 Results

### 3.1 Sample Description

Demographic and clinical characteristics at baseline for the full sample as well as the respective subsamples with FSS and CBQ data at the first visit are summarized in Table 1. At baseline, patients had an average of M = 1.93 diagnoses (SD = 0.71, Range: 1–5). Most frequent diagnoses were depression (93.25%), anxiety disorders (66.25%), with other diagnoses being PTSD (8.16%), ADHD (6.75%), bipolar disorder (4.87%), eating disorder (2.67%), and substance abuse (1.57%). Further, 6.59% had other diagnoses and for 1.73%, diagnostic information was missing.

**TABLE 1:** Sample description including demographic data and baseline suicidality prevalence.

	Total sample	FSS subsample	CBQ subsample	Difference between subsamples
Patients K	637	393	244	
Number of assessments *N*	3791	2253	1538	
IOP treatment duration in days	M = 36.52; SD = 14.46; Range: 1–63	M = 37.28; SD = 13.84; Range: 1–63	M = 35.31; SD = 13.35; Range: 2–63	*d* = −0.134
Average Compliance (100% = 1 self‐report per treatment‐week)	M = 120.82% SD = 38.55	M = 113.50% SD = 36.79	M = 134.56% SD = 41.57	*d* = 0.544
Demographics				
Age	M = 15.83; SD = 1.44	M = 15.77, SD = 1.45	M = 15.92, SD = 1.40	*d* = 0.105
Sex at birth				
Female	76.61%	79.13%	72.54%	OR = 1.44
Male	23.39%	20.87%	27.46%	
Gender Identity				
Cis male/female	88.85%	82.19%	99.59%	OR = 0.019 (Cis vs. all other)
Trans/trans‐male/trans‐female	5.02%	7.89%	0.41%	
Queer/Nonbinary/Gender‐fluid/Other	6.12%	9.92%	—	
Ethnicity				
White	82.57%	83.72%	80.74%	OR = 1.23 (white vs. other)
African American	8.01%	7.12%	9.43%	
Hispanic	2.83%	2.04%	4.10%	
Asian	1.88%	2.04%	1.64%	
Mixed	2.51%	2.29%	2.87%	
NA	2.20%	2.80%	1.23%	
Baseline prevalence STBs (range 0–5, respectively)				
Suicidal ideation at baseline	M = 3.74, SD = 1.22	M = 3.84, SD = 1.19	M = 3.57, SD = 1.28	*d* = −0.220
History of suicidal ideation	M = 2.02; SD = 1.69	M = 2.25; SD = 1.73	M = 1.64; SD = 1.55	*d* = −0.367
Suicidal behavior at baseline	M = 3.98; SD = 1.14	M = 4.05; SD = 1.08	M = 3.87; SD = 1.22	*d* = −0.159
History of suicidal behavior	M = 2.36; SD = 1.72	M = 2.55; SD = 1.73	M = 2.04; SD = 1.66	*d* = −0.299
Number of comorbid diagnoses	M = 1.93; SD = 0.71	M = 1.96; SD = 0.71	M = 1.89; SD = 0.71	*d* = −0.099

*Note:* Total sample = all patients with at least one valid weekly assessment with sufficient outcome data, FSS subsample = all patients with at least one valid weekly assessment with sufficient outcome data and FSS assessment in the first week, CBQ subsample = all patients with at least one valid weekly assessment with sufficient outcome data and CBQ assessment in the first week.

Length of IOP varied considerably, with an average treatment duration of M = 5.21 weeks (SD = 2.07). Due to the between‐person variation in treatment duration and the variability in treatment intervals, the reference point for compliance calculation was set to 1 assessment per individual treatment week. On average, participants answered more than once per week, resulting in an overall compliance rate of 120% (see Table 1). For 316 participants (49.61%), additional treatment (in the form of another IOP treatment, maintenance outpatient sessions, etc.) was recorded following the initial IOP.

### 3.2 Symptom Trajectories Over the Course of Treatment

**FIGURE 1:**
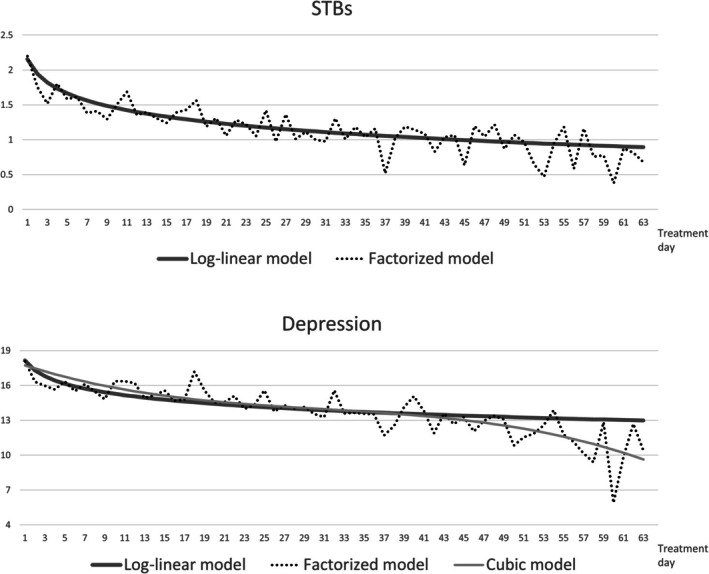
Comparison of best fitting estimated treatment trajectories for STBs and depression with factorized point‐estimates for each day of treatment.

For STBs, the best fitting model according to low AIC and BIC was a model with a log‐linear association (see Table 2 for an overview of AICs and BICs by model). Random‐intercept multilevel regressions of STBs showed significant logarithmic improvement over time (*b* = −0.305, SE = 0.011, T = −28.10, *p* < 0.001) with the estimated trajectory fitting well to the distribution of factorized point estimates for each treatment (see Figure 1, upper part). For depression scores, the best fitting model was a model with linear, quadratic, and cubic predictors, closely followed by the log‐linear model (ΔBIC = 1). Visual inspection of the two competing models (see Figure 1, lower part) shows that the cubic model better fits the factorized point estimates at the late stages, that is, 8th and 9th week of treatment. Comparing models for a subset of the first 7 treatment weeks showed the log‐linear model to be the best fitting (AIC = 21,785, BIC = 21,822 vs. cubic model: AIC = 21,782, BIC = 21,807). Since the difference in fit between the log‐linear and cubic models was comparatively small, the log‐linear model was the more parsimonious, and since differences in fit seemed to be localized to the last 2 weeks of treatment, with only 18.52% of the sample having a recorded treatment epoch of more than 49 days of consecutive IOP, we decided to use the log‐linear model for all outcomes in subsequent moderator analysis.

**TABLE 2:** Comparison of model fit for association between session number and different outcome measures.

Model type	Outcome											
	ASQ Sum				Depression				Depression (Day 1–49 subsample)			
	logLik	AIC	BIC	Deviance	logLik	AIC	BIC	Deviance	logLik	AIC	BIC	Deviance
Linear association	−5267	10,542	10,567	10,534	−11,286	22,580	22,605	22,572	−10,906	21,820	21,845	21,812
Logarithmic association	−5164	**10,336**	**10,360**	10,328	−11,276	22,560	22,585	22,552	−10,887	**21,782**	**21,807**	21,774
Linear + quadratic association	−5208	10,426	10,457	10,416	−11,279	22,568	22,599	22,558	−10,894	21,798	21,829	21,788
Linear + quadratic + cubic linear + quadratic association	−5181	10,373	10,411	10,361	−11,267	22,547	22,584	22,535	−10,887	21,785	21,822	21,773

*Note:* The AIC‐ and BIC‐score in all columns denoting the best fitting model is printed in bold.

### 3.3 Moderation of Symptom Trajectories by Baseline Family Factors

Random‐intercept, fixed‐slope multilevel regression analysis testing for the interaction of intervention week (i.e., session count) and family factors (i.e., FSS‐ or CBQ‐sum‐score) are summarized in Table 3. As can be seen, higher family cohesion (FSS‐scores) at the beginning of IOP was significantly associated with lower depression severity at the beginning. Moreover, an interaction effect of FSS and treatment day on weekly STBs scores was significant and indicated more pronounced improvement when family cohesion was higher at the start of the intervention. This effect was also found for suicidal thoughts (*β* = −0.11, *p* = 0.014), but not for logistic regression of suicidal behavior presence (OR = 0.99, *p* = 0.014 see Table S1 full estimate statistics). Regarding conflict behavior, we found a main effect on STBs, surprisingly showing that higher levels of family conflict were associated with lower baseline STBs. Additionally, there was a tendency towards less improvement in STBs over time as a function of higher conflict levels, but this interaction effect did not reach statistical significance (*β* = 0.09, *p* = 0.093). When only suicidal thoughts were predicted, the interaction was significant (*β* = 0.10, *p* = 0.039), whereas the interaction was non‐significant for the suicidal behaviors outcome (OR = 0.99, *p* = 0.103, see Table S1).

**TABLE 3:** Multilevel regression models predicting Suicidality/depression (outcome) by the predictors treatment day (log‐transformed) × baseline family factor (FSS or CBQ).

Model outcome	Family factor	Patients	Assess.	Main effect week number (log)				Main effect family factor variable				Interaction effect			
		*k*	*n*	*β*	*B*	SE	*p*	*β*	*B*	SE	*p*	*β*	*B*	SE	*p*
STBs	—	637	3791	**−0.46**	**−0.305**	0.011	< 0.001	—	—	—	—	—	—	—	—
Depression	—	637	3713	**−0.36**	**−1.246**	0.055	< 0.001	—	—	—	—	—	—	—	—
STBs	FSS	393	2253	**−0.49**	**−0.350**	0.014	< 0.001	0.06	0.004	0.005	0.714	**−0.11**	**−0.004**	0.002	0.008
Depression	FSS	393	2209	**−0.42**	**−1.437**	0.069	< 0.001	**−0.20**	**−0.115**	0.037	0.002	0.04	0.008	0.008	0.345
STBs	CBQ	244	1538	**−0.33**	**−0.231**	0.018	< 0.001	**−0.27**	**−0.031**	0.011	0.007	0.09	0.005	0.003	0.093
Depression	QBQ	244	1504	**−0.27**	**−0.954**	0.091	< 0.001	0.12	0.108	0.071	0.861	0.01	0.003	0.016	0.861

*Note:* Estimates printed in bold denote effects with *p* < 0.05.

### 3.4 Heterogeneity in Family Factor Moderation Effects

An overview of all tests of three‐way interaction effects including treatment day × one of the family factor variables × a demographic/clinical variable is shown in Table 4. The pattern of effects remained comparable in terms of effect sizes when suicidal thoughts were the dependent variable instead of the 4‐item sum score, but only the effect for minority status remained significant (see Table S2). Logistic regression models predicting suicidal behavior presence could not be estimated, likely due to the combination of model complexity and low occurrence of suicidal behaviors (194 reported cases of suicidal behaviors, 5.1% of all assessments, with 113 of them reported at baseline).

**TABLE 4:** Tests for putative demographic/clinical moderators of treatment × family factor effect (three‐way interactions of separate multilevel models, respectively).

Moderator	Outcome	Three way interaction of moderator × treatment day × …							
		… FSS scores				… CBQ scores			
		*β*	*b*	SE	*p*	*β*	*b*	SE	*p*
Male sex at birth	STBs	−0.02	−0.002	0.004	0.682	0.03	0.004	0.008	0.636
	Depression	−0.02	−0.008	0.021	0.715	0.06	0.037	0.039	0.339
Non‐cis gender identity	STBs	0.08	0.007	0.006	0.220	—	—	—	—
	Depression	0.06	0.028	0.029	0.328	—	—	—	—
Age	STBs	−0.03	−0.001	0.001	0.417	0.06	0.003	0.002	0.25
	Depression	0.00	−0.001	0.006	0.919	**0.15**	**0.031**	0.012	0.007
Ethnically minoritized (vs. white)	STBs	**0.16**	**0.017**	0.005	< 0.001	**−0.14**	**−0.021**	0.009	0.014
	Depression	0.09	0.047	0.024	0.063	0.04	−0.029	0.044	0.512
Suicidal ideation levels at baseline	STBs	−0.01	0.000	0.001	0.824	−0.07	−0.003	0.003	0.232
	Depression	0.03	0.005	0.006	0.443	0.01	0.003	0.013	0.828
Suicidal behavior levels at baseline	STBs	−0.02	0.000	0.001	0.851	0.04	0.001	0.002	0.751
	Depression	0.13	0.005	0.005	0.255	0.05	0.004	0.011	0.711
Most severe suicidal ideation	STBs	**−0.09**	**−0.003**	0.001	0.030	0.02	0.001	0.003	0.719
	Depression	−0.01	−0.002	0.007	0.771	−0.01	−0.002	0.014	0.895
Most severe suicidal behavior	STBs	−0.08	−0.002	0.001	0.061	0.07	0.002	0.002	0.200
	Depression	−0.01	−0.001	0.005	0.849	−0.01	−0.002	0.010	0.863
No. of comorbid diagnoses	STBs	0.05	0.003	0.002	0.251	**−0.11**	**−0.011**	0.005	0.021
	Depression	0.05	0.014	0.012	0.254	−0.01	−0.014	0.025	0.085

*Note:* Estimates printed in bold denote significant effects with *p* < 0.05.

**FIGURE 2:**
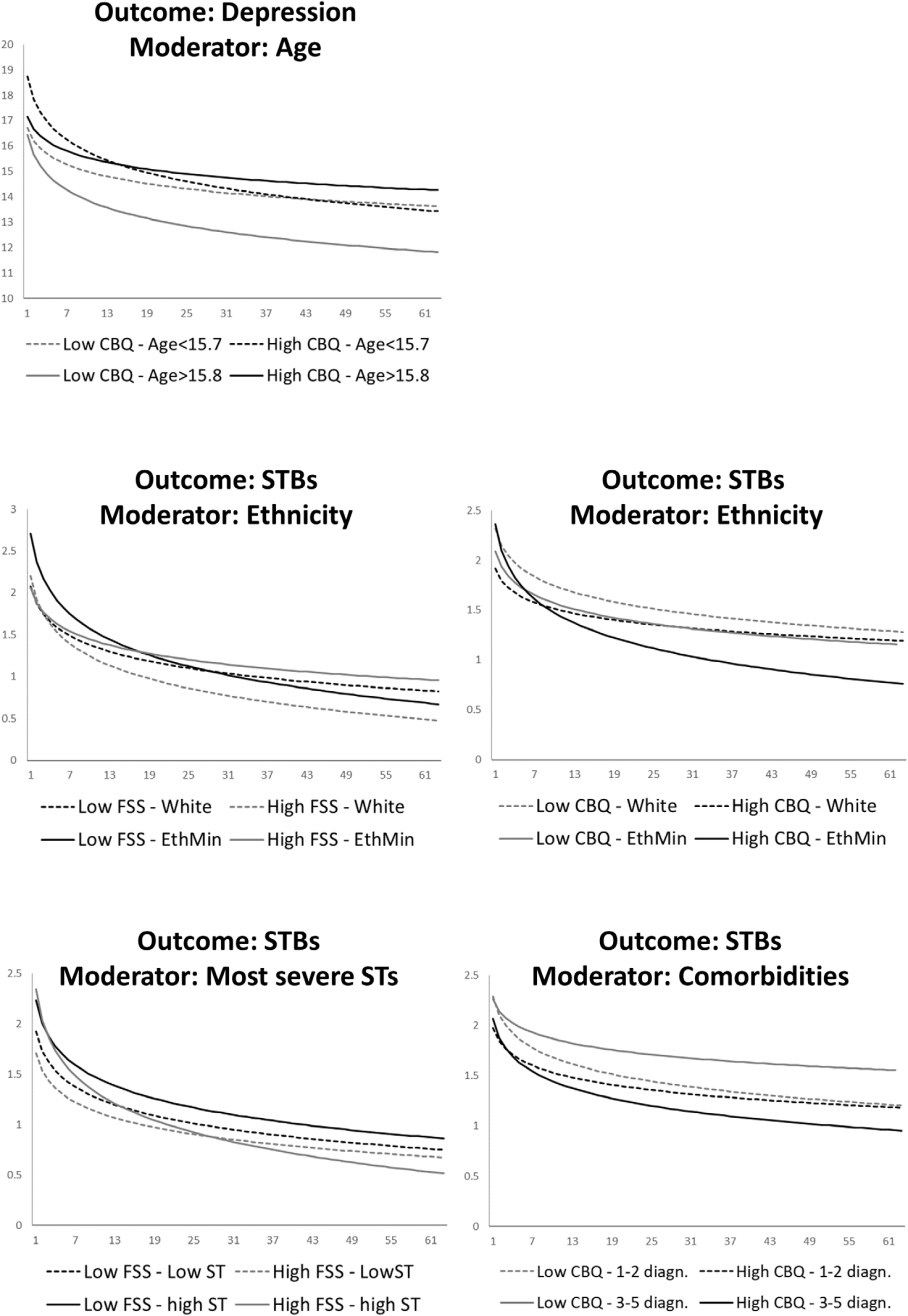
Overview of significant baseline moderation effects including treatment day × one of the family factor moderators × a demographic/clinical variable.

As can be seen, five significant moderation effects emerged. By visual inspection (see Figure 2), different cluster of interactions could be observed: (1) for the interaction effect involving patient age and conflict behavior on depression levels (Figure 2, top row), younger participants started with higher depression severity when conflict behavior was high but improved up to reaching roughly the same endpoint levels as younger patients who started with low family conflict levels. By contrast, for older youth, those who rated high family conflict demonstrated reduced improvement over time, whereas older, low family conflict patients showed the highest improvement rate in depression severity. Regarding (2) the significant interaction effects involving ethnic minority status (Figure 2, mid row), less positive family factors at baseline (i.e., low FSS, high CBQ) in minoritized patients were surprisingly associated with more improvement over time, whereas white participants showed slightly more average improvement over time, when conflict levels (CBQ) were low and family cohesion was high. Finally, (3) for patients who showed more severe lifetime levels of suicidal thoughts, high baseline family cohesion was associated with a more positive outcome in STBs over time and the largest reduction in STBs. By contrast, for patients with less severe lifetime levels of suicidal thoughts, differences in average treatment response over time due to baseline family cohesion were far less pronounced. Contrary to this the interaction effect involving family conflict and number of diagnoses showed the opposite pattern, with larger differences between the high‐conflict groups and highest improvements in those participants with multiple diagnoses and high baseline family conflict.

## 4 Discussion

In this study, we tested the influence of established family factors, namely cohesion and conflict, on the trajectory and outcome of outpatient treatment for STBs in a sample of youth at high risk for death by suicide. In part, our findings are consistent with previous research indicating that unfavorable family risk and resilience factors are associated with more severe starting levels of pathology and poorer therapy outcomes (Abbott et al. 2019; King et al. 1997). The only seemingly paradoxical effect pertained to a main effect of family conflict, which was associated with less STBs at baseline when family conflict levels were higher. Possibly, higher conflict at baseline coincides with a general (albeit not necessarily completely functional) tendency to actively engage perceived problems and more self‐efficacy (vs. hopelessness and passivity), which in turn co‐varies with less severe symptoms.

Of importance, however, most of the tested main or interaction effects of family factors were non‐significant in the main analysis. Taken together with the consistently significant effects of treatment over time, this constitutes a quite promising result because, on average, patients improved over the course of the IOP irrespective of familial resources or familial risk factors at the start of the treatment. Overall, the IOP seems to show the desired effects even in the face of further complicating family problems.

While the overall main effect of the treatment remained stable and consistently showed improvement across all analyses, further analyses of the heterogeneity of the effects of family factors revealed some potential gradual differences in the degree of treatment response that warrant further discussion. First, the effect of family conflict seems to vary by patient age, with older adolescents showing reduced and lowest overall improvement when family conflicts are more severe. Existing developmental research showed that over the course of healthy normative development, conflict levels peak at mid‐adolescence (Laursen et al. 1998), prior to the age of 16. Simultaneously, other trajectories associated with more distress have been shown to be defined by persisting/increasing conflict levels in late adolescence (Koepke and Denissen 2012). Possibly, the convergence of individuation processes that become more prevalent at ages of 16–18 (Koepke and Denissen 2012) and persisting familial conflicts mutually reinforce each other and impair improvement. An in‐depth analysis, for example, in the form of an ecological momentary assessment exploring the dynamic interactions of family conflict occurrence, dysfunctional self‐schema activation, and state symptom levels in this group could reveal globally and individually relevant treatment targets for adjunct intervention modules to improve the overall response to outpatient treatment programs.

Second, some of the significant three‐way interaction effects tentatively suggest that family cohesion constitutes a more decisive factor in more severe cases of suicidality, whereas conflict levels are more relevant in patients with complex multimorbidity.

Finally, the impact of family cohesion and conflict on treatment success appears to vary between ethnic majority versus minoritized groups. Notably, minoritized patients showed greater improvement when baseline family cohesion was low and family conflict levels were high. Prior research has shown that the cross‐sectional association between STBs in youths and family conflict levels is increased in some minoritized groups (Assari et al. 2021), which would indicate that they show more conflict/less cohesion given the same level of STB at the start of the treatment. Conceivably, early treatment gains in pathology might enhance family cohesion, boosting perceived belonging and further reducing STBs (Joe et al. 2018). Future studies should examine how family dynamics evolve during treatment and whether these changes reinforce a positive therapeutic trajectory, particularly in ethnically minoritized youth (Brown et al. 2024).

Taken together, our results suggest the existence of adolescent patient groups, for whom baseline family factors influence therapy progress in different ways. This result warrants further investigation in naturalistic settings to explore whether such subgroups can be identified ex ante or in early stages of the treatment to allow for a differential use of family factor information according to their relevance to the current case when planning and optimizing treatment. Further, to effectively use the moderation effects we found for treatment optimization, a better understanding of the mechanism underlying the found moderation effects is needed. A potential explanation for the aforementioned, seemingly paradoxical effects could be that IOP, although not targeting family dynamics in its core components, leads to changes in the respective family factors (i.e., ameliorates risk and fosters resilience factors). This could in turn positively influences subsequent symptomatic improvements. For inverse effects similar to those found in prior studies (Zisk et al. 2019), this appears to be especially likely, since those treatments targeted family and family relationships. For these treatments, patients with more intra‐familial problems at baseline start with more potential room for improvement. Regarding multifaceted treatments such as our IOP, future iterations of this study could test this hypothesis by documenting attendance of optional multi‐family‐group components and exploring whether more improvement in patients with high conflict/low‐cohesion families is tied to the amount of family focused treatment units received. Moreover, one recent trial includes daily assessment of family conflict and depression symptoms (Miklowitz et al. 2021), which would allow for modeling and testing co‐developmental trajectories of family factors and symptoms over the course of therapy. Such studies with more detailed, fine‐grained assessments could provide crucial information about the dynamic changes in family factors in order to fully understand how they impact symptomatic improvement. Next to the family‐factor‐improvement hypothesis described above, another possible explanation would be that qualitative changes in the appraisal of family factors occur during therapy. This could also explain the pattern of results such as the inverse association between baseline family factors and subsequently higher improvements in ethnically minoritized patients. Ideally, future therapy process studies include an extensive list of family factors which are consistently measured from multiple perspectives over the full course of the treatment in order to explore the temporal dynamics of change in family factors and their effect on therapy progress in greater detail.

### 4.1 Strengths and Limitations

Strengths of this study are the longitudinal design with high levels of compliance regarding the weekly assessments. Further, the naturalistic, data‐driven variation in therapy length until improvement in STBs were achieved strengthens the generalizability of our results to clinical practice. At the same time, some limiting factors need to be considered. The assessment of family factors was relatively simple and relied solely on self‐report by the adolescent patients. Consequently, possible confounding third variables that can explain our results need to be considered. In particular, we cannot exclude that both initial family factor ratings and symptom ratings over time are influenced by habitual patient cognition (e.g., more hopeful or optimistic patients could initially self‐rate their familial situation higher and then tend to notice and subsequently report improvements faster). A more differentiated assessment of family cohesion and conflict aspects that includes adolescent and parent perspectives as well as ‐ideally‐ behavioral measures could provide a more complete and valid picture. The two subsamples used for the analyses of family cohesion and family conflict effects showed overall only small differences in demographic and baseline clinical variables. The only exception was gender identity: In the family conflict subsample transgender or nonbinary participants were not represented, so this difference could factor into any diverging findings between family cohesion and family conflict. Moreover, our results regarding suicidal behaviors are somewhat limited due to more precise assessment methods for suicidal thoughts and plans. However, within the context of this IOP, re‐occurrence of suicidal behaviors was very rare, so this focus on thoughts and plans may fit to the general scope of changes and fluctuations in outpatient treatment. Finally, our research on the moderating effects of family factors continued the tradition of secondary analyses of therapy trials. Whereas this study was not a secondary analysis of an RCT with narrowly defined intervention content and a fixed duration of the intervention to increase the validity of the efficacy test an RCT aims for, family factors were still not the main focus of the assessment, leading at the very least to assessment of family factors for only a subset of the participants.

## 5 Conclusion

In sum, this study tentatively suggests that family factors at the start of treatment modulate the effect of psychotherapy on STBs in particular subgroups of youths at high risk for death by suicide, partially influencing—but rarely fully negating—effects of intensive outpatient treatment. Future studies need to further elucidate how family factors might change over the course of treatment and how these changes co‐vary with and influence subsequent changes in STBs in order to utilize family factors as a potential treatment target to further optimize individualized treatments.

## Funding

The clinical study for this research has been funded by State of Pennsylvania Appropriation Funds.

## Ethics Statement

All study procedures were approved by the University of Pittsburgh Institutional Review Board (Protocol Number: 19030078).

## Consent

Participants and a parent/legal guardian provided written informed assent/consent for their clinical data to be used for research purposes in order to be included in this analysis.

## Conflicts of Interest

Dr. David Brent has received research support from the American Foundation for Suicide Prevention, the National Institute of Mental Health, the Once Upon a Time Foundation, and the Beckwith Foundation, royalties from Guilford Press, from the electronic self‐rated version of the C‐SSRS from eRT Inc., and from performing duties as a UptoDate Psychiatry Section Editor, and consulting fees from Healthwise. Dr. Tina R. Goldstein has received research support from the National Institute of Mental Health, the Substance Abuse and Mental Health Services Administration, and The Pittsburgh Foundation, The Beckwith Foundation, and royalties from Guilford Press. Ms. Kimberly Poling has received royalties from Guilford Press. The other authors declare no conflicts of interest.

## Supporting information


**Table S1:** Multilevel regression models predicting Suicidality sum scores vs. suicidal thoughts sum‐scores vs. suicidal behavior (Outcome) by the predictors treatment day (log‐transformed) × baseline family factor (FSS or CBQ).
**Table S2:** Tests for putative demographic/clinical moderators of treatment × family factor effect (three‐way interactions of separate multilevel models, respectively) for the different suicidality outcomes Suicidality sum scores (STBs) vs. suicidal thoughts sum‐scores vs. suicidal behavior.

## Data Availability

The data are not publicly available due to privacy or ethical restrictions. Per the University of Pittsburgh Institutional Review Board, the deidentified dataset may be requested via an Honest Broker system (contact: Giovanna Porta, MS, portgx@upmc.edu).
