# Supplementary material for: The Impact of Family Factors on Adolescent Intensive Outpatient Psychotherapy Outcomes for Suicidal Thoughts, Behaviors, and Depression
Source: Suicide Life Threat Behav. 2026 May 5;56:e70104. doi: 10.1111/sltb.70104 (PMC13141666; doi:10.1111/sltb.70104)
Supplement: Supplementary file 1 — Table S1: Multilevel regression models predicting Suicidality sum scores vs. suicidal thoughts sum‐scores vs. suicidal behavior (Outcome) by the predictors treatment day (log‐transformed) × baseline family factor (FSS or CBQ). Table S2: Tests for putative demographic/clinical moderators of treatment × family factor effect (three‐way interactions of separate multilevel models, respectively) for the different suicidality outcomes Suicidality sum scores (STBs) vs. suicidal thoughts sum‐scores vs. suicidal behavior. [file SLTB-56-0-s001.docx]

**Supplemental Table S1**

Multilevel regression models predicting Suicidality sum scores vs. suicidal thoughts sum-scores vs. suicidal behavior (Outcome) by the predictors treatment day (log-transformed) x baseline family factor (FSS or CBQ)

| Model Outcome | Family factor | Main effect week number (log) | | | | Main effect family factor variable | | | | Interaction effect | | | |
| --- | --- | --- | --- | --- | --- | --- | --- | --- | --- | --- | --- | --- | --- |
|  |  | β/OR | B | SE | p | β/OR | B | SE | p | β/OR | B | SE | p |
| STBs_sum | - | **-0.46** | **-0.305** | **0.011** | **<0.001** | - | - | - | - | - | - | - | - |
| Suicidal thoughts | - | **-0.70** | **-0.260** | **0.010** | **<0.001** | - | - | - | - | - | - | - | - |
| Suicidal behavior (OR) | - | NA – model failed to converge | | | |  |  |  |  |  |  |  |  |
| STBs | FSS | **-0.49** | **-0.350** | **0.014** | **<0.001** | 0.06 | 0.004 | 0.005 | 0.714 | **-0.11** | **-0.004** | **0.002** | **0.008** |
| Suicidal thoughts | FSS | **-0.83** | **-0.310** | **0.012** | **<0.001** | 0.00 | 0.002 | 0.005 | 0.716 | **-0.09** | **-0.004** | **0.001** | **0.014** |
| Suicidal behavior (OR) | FSS | **0.39** | **-0.997** | **0.105** | **<0.001** | 1.01 | 0.013 | 0.020 | 0.524 | 0.99 | -0.013 | 0.01 | 0.258 |
| STBs | CBQ | **-0.33** | **-0.231** | **0.018** | **<0.001** | **-0.27** | **-0.031** | **0.011** | **0.007** | 0.09 | 0.005 | 0.003 | 0.093 |
| Suicidal thoughts | CBQ | **-0.51** | **-0.180** | **0.016** | **<0.001** | **-0.16** | **-0.030** | **0.011** | **0.004** | **0.10** | **0.006** | **0.003** | **0.039** |
| Suicidal behavior (OR) | CBQ | **0.26** | **-1.347** | **0.165** | **<0.001** | 1.01 | 0.011 | 0.070 | 0.879 | 0.95 | -0.048 | 0.029 | 0.103 |

Note: estimates printed in bold denote effects with p<0.05. For some models, Logistic Regression could not be calculated due to lack of model convergence. In these cases, the respective rows show “NA – model failed to converge“

**Supplemental Table S2**

Tests for putative demographic/clinical moderators of treatment x family factor effect (three-way interactions of separate multilevel models, respectively) for the different suicidality outcomes Suicidality sum scores (STBs) vs. suicidal thoughts sum-scores vs. suicidal behavior

| Moderator | Outcome | Three way interaction of moderator x treatment day x … | | | | | | | | |
| --- | --- | --- | --- | --- | --- | --- | --- | --- | --- | --- |
|  |  | … FSS scores | | | | | … CBQ scores | | | |
|  |  | β/OR | b | SE | p | β/OR | | b | SE | p |
| Male sex at birth | STBs | -0.02 | -0.002 | 0.004 | 0.682 | 0.03 | | 0.004 | 0.008 | 0.636 |
|  | Suic. thoughts | -0.06 | -0.000 | 0.004 | 0.986 | 0.16 | | 0.011 | 0.008 | 0.108 |
|  | Suic. Behav. (OR) | NA model failed to converge | | | | NA model failed to converge | | | | |
| Non-cis gender identity | STBs | 0.08 | 0.007 | 0.006 | 0.220 | - | | - | - | - |
|  | Suic. thoughts | 0.15 | 0.007 | 0.004 | 0.093 | - | | - | - | - |
|  | Suic. Behav. (OR) | NA model failed to converge | | | | - | | - | - | - |
| Age | STBs | -0.03 | -0.001 | 0.001 | 0.417 | 0.06 | | 0.003 | 0.002 | 0.250 |
|  | Suic. thoughts | -0.06 | -0.002 | 0.001 | 0.071 | 0.09 | | 0.003 | 0.002 | 0.100 |
|  | Suic. Behav. (OR) | NA model failed to converge | | | | NA model failed to converge | | | | |
| Ethnically minoritized (vs. white) | STBs | **0.16** | **0.017** | **0.005** | **<0.001** | **-0.14** | | **-0.021** | **0.009** | **0.014** |
|  | Suic. thoughts | **0.35** | **0.014** | **0.004** | **0.001** | -0.22 | | -0.013 | 0.008 | 0.091 |
|  | Suic. Behav. (OR) | 1.02 | 0.028 | 0.025 | 0.270 | NA model failed to converge | | | | |
| Suicidal ideation levels at baseline | STBs | -0.01 | 0.000 | 0.001 | 0.824 | -0.07 | | -0.003 | 0.003 | 0.232 |
|  | Suic. thoughts | -0.03 | -0.001 | 0.001 | 0.603 | -0.06 | | -0.003 | 0.002 | 0.270 |
|  | Suic. Behav. (OR) | NA model failed to converge | | | | NA model failed to converge | | | | |
| Suicidal behavior levels at baseline | STBs | -0.02 | 0.000 | 0.001 | 0.851 | 0.04 | | 0.001 | 0.002 | 0.751 |
|  | Suic. thoughts | -0.01 | 0.000 | 0.001 | 0.821 | -0.01 | | 0.000 | 0.002 | 0.979 |
|  | Suic. Behav. (OR) | NA model failed to converge | | | | NA model failed to converge | | | | |

| Moderator | Outcome | Three way interaction of moderator x treatment day x … | | | | | | | | |
| --- | --- | --- | --- | --- | --- | --- | --- | --- | --- | --- |
|  |  | … FSS scores | | | | | … CBQ scores | | | |
|  |  | β/OR | b | SE | p | β/OR | | b | SE | p |
| Most severe suicidal ideation | STBs | **-0.09** | **-0.003** | **0.001** | **0.030** | 0.02 | | 0.001 | 0.003 | 0.719 |
|  | Suic. thoughts | -0.06 | -0.002 | 0.001 | 0.063 | <0.01 | | 0.001 | 0.002 | 0.452 |
|  | Suic. Behav. (OR) | NA model failed to converge | | | | NA model failed to converge | | | | |
| Most severe suicidal behavior | STBs | -0.08 | -0.002 | 0.001 | 0.061 | 0.07 | | 0.002 | 0.002 | 0.200 |
|  | Suic. thoughts | -0.07 | -0.002 | 0.001 | 0.075 | 0.01 | | 0.001 | 0.002 | 0.452 |
|  | Suic. Behav. (OR) | NA model failed to converge | | | | NA model failed to converge | | | | |
| Number of comorbid diagnoses | STBs | 0.05 | 0.003 | 0.002 | 0.251 | **-0.11** | | **-0.011** | **0.005** | **0.021** |
|  | Suic. thoughts | 0.05 | 0.002 | 0.002 | 0.268 | -0.08 | | -0.006 | 0.004 | 0.147 |
|  | Suic. Behav. (OR) | NA model failed to converge | | | | NA model failed to converge | | | | |

Note: estimates printed in bold denote effects with p<0.05.
For some models, Logistic Regression could not be calculated due to lack of model convergence. In these cases, the respective rows show “NA – model failed to converge“
